# CrWRKY57 and CrABF3 cooperatively activate *CrCYCD6;1* to modulate drought tolerance and root development

**DOI:** 10.1093/hr/uhaf158

**Published:** 2025-06-20

**Authors:** Jinxia Mo, Xinting Xiong, Zaofa Zhong, Lu Liu, Ying Xiong, Min Wang, Wenshan Dai, Shaohua Zeng, Ting Peng

**Affiliations:** National Navel Orange Engineering Research Center, College of Life Sciences, Gannan Normal University, Shida South Road, Rongjiang New District, Ganzhou 341000, China; National Navel Orange Engineering Research Center, College of Life Sciences, Gannan Normal University, Shida South Road, Rongjiang New District, Ganzhou 341000, China; National Navel Orange Engineering Research Center, College of Life Sciences, Gannan Normal University, Shida South Road, Rongjiang New District, Ganzhou 341000, China; National Navel Orange Engineering Research Center, College of Life Sciences, Gannan Normal University, Shida South Road, Rongjiang New District, Ganzhou 341000, China; National Navel Orange Engineering Research Center, College of Life Sciences, Gannan Normal University, Shida South Road, Rongjiang New District, Ganzhou 341000, China; National Navel Orange Engineering Research Center, College of Life Sciences, Gannan Normal University, Shida South Road, Rongjiang New District, Ganzhou 341000, China; National Navel Orange Engineering Research Center, College of Life Sciences, Gannan Normal University, Shida South Road, Rongjiang New District, Ganzhou 341000, China; National Navel Orange Engineering Research Center, College of Life Sciences, Gannan Normal University, Shida South Road, Rongjiang New District, Ganzhou 341000, China; Guangdong Provincial Key Laboratory of Applied Botany, South China Botanical Garden, Chinese Academy of Sciences, Xingke Road 723, Tianhe District, Guangzhou 510650, China; National Navel Orange Engineering Research Center, College of Life Sciences, Gannan Normal University, Shida South Road, Rongjiang New District, Ganzhou 341000, China

## Abstract

Drought is a major abiotic stress. WRKYs are one of the largest families of transcription factors (TFs) in plants. The effects of most WRKYs on developmental regulation and drought adaptation in *Citrus* remain largely unclear. *Citrus reticulata* cv. Sanhu hongju (Sanhu) is a drought-tolerant variety from Jiangxi Province, China. Here, we report a differentially expressed *CrWRKY57* gene in drought-treated Sanhu leaves through transcriptome analysis. Its transcriptional expression could be induced by abscisic acid (ABA) treatment and water deficit. Overexpression of *CrWRKY57* in lemon (*Citrus limon*) and tobacco (*Nicotiana tabacum*) confers enhanced drought tolerance, while RNA interference (RNAi)-mediated silencing in Sanhu increases dehydration susceptibility and reduces root volume. Moreover, virus-induced gene silencing-mediated knockdown of *CrWRKY57* in Sanhu reduces primary root length and lateral root number by nearly 50% compared to the control. The results of yeast two-hybrid, co-immunoprecipitation assays and bimolecular fluorescence complementation demonstrate that CrWRKY57 interacts with CrABF3, a key TF in ABA signaling. Silencing *ClABF3*, its homolog in lemon, also increases drought sensitivity and disrupts root system development. Together, CrWRKY57 and CrABF3 directly activate the promoter of the cell cycle gene *CrCYCD6;1* by binding to W-box and ABRE elements, respectively. Furthermore, silencing *CrCYCD6;1* in Sanhu also severely reduces primary root length and lateral root number. Collectively, our findings provide a new perspective of *CrWRKY57* as a positive player in drought response and highlight the role of the CrWRKY57-CrABF-*CrCYCD6;1* module in enhancing drought tolerance by modulating root development.

## Introduction

The increasing frequency of climate change leads to more severe and long-lasting droughts [1, 2]. Although agricultural crops face multiple biotic and abiotic stresses, annual productivity losses caused by drought exceed those attributable to all pathogenic factors combined [3]. Therefore, enhancing drought tolerance of crops represents a critical strategy for ensuring future food security. Consequently, it is imperative to identify key genetic determinants and elucidate the underlying regulatory mechanisms in drought response.

Plants’ drought adaptation involves multiple physiological responses, such as root architecture remodeling for enhanced water uptake, abscisic acid (ABA)-mediated stomatal closure, enzymatic and nonenzymatic reactive oxygen species (ROS) quenching systems, and osmoprotectants accumulation for membrane integrity protection [4–8]. Among these adaptive strategies, transcription factors (TFs) serve as central molecular switches that coordinately activate or repress downstream target genes, orchestrating an integrated stress response that improves whole-plant drought tolerance [9]. Functioning as plant-specific master regulators, WRKY TFs operate as a critical signaling hub that fine-tunes the interplay between stress-responsive pathways and developmental programs [10, 11]. The WRKY domain consists of 60-amino acid N-terminus region, which contains the signature WRKYGQK heptapeptide sequence, followed by a zinc-finger motif with either a CX4-5CX22-23HXH or CX7CX23HXC configuration [12]. WRKY TFs specifically recognize and bind to the W-box (TTGAC[T/C]) *cis*-regulatory element within promoter regions of target genes, thereby fulfilling their transcriptional regulatory functions [10]. WRKYs are categorized into three major groups (I–III) based on both the number of WRKY domains and zinc-finger configuration, with Group II subdivided into five distinct subgroups (IIa–IIe) [12]. However, this grouping shows limited correlation with functional roles [11]. Over recent decades, extensive studies have been performed to establish WRKYs as important regulators in ABA signaling and stress responses [12, 13]. According to the public availability of *Citrus* genomic and transcriptomic data, 100 WRKY family members from *Citrus* species (*Citrus sinensis*, *Citrus clementina*, and *Citrus unshiu*) were identified, representing promising genetic targets for *Citrus* cultivar improvement [14]. In *Fortunella crassifolia* (a close relative of *Citrus*), FcWRKY70 enhances drought tolerance by modulating putrescine synthesis [15]. *FcWRKY40* enhances osmotic stress tolerance by orchestrating ion homeostasis and proline accumulation through direct transcriptional activation of genes encoding salt overly sensitive 2 and △-1-pyrroline-5-carboxylate synthetase 1 [16]. Further investigations are needed to provide more insights into the regulatory hierarchy of WRKY-mediated drought tolerance in *Citrus*.

ABA acts as an essential orchestrator of the above-mentioned physiological changes and TF-mediated regulatory network in plant drought response [17–20]. Under drought conditions, ABA binds to the pyrabactin resistance (PYR)/PYR1-LIKE76 (PYL)/regulatory components of ABA receptor (RCAR), which then recruit and inhibit clade A type 2C phosphatases (PP2Cs), resulting in the formation of the PYR/PYL/RCAR-ABA-PP2C signaling complex [21, 22]. Consequently, the SNF1-related protein kinase family (SnRKs) is released from the inhibition of PP2Cs, enabling SnRKs-mediated phosphorylation and activation of downstream targets, such as the ABRE-binding (AREB) proteins or ABA-response element (ABRE)-binding factors (ABFs) [2, 23–25]. For example, in *Poncirus trifoliata* (L.) Raf. (a deciduous close relative of evergreen *Citrus*), PtrSnRK2.4-mediated phosphorylation of PtrABF2 enhances drought tolerance by regulating *arginine decarboxylase*-mediated putrescine biosynthesis [26]. In *Arabidopsis*, all four ABFs function as master regulators that transduce ABA-mediated signals and activate downstream gene networks to confer drought enhancement [24, 27]. However, emerging evidence suggests ABFs likely function in concert with complementary TFs to activate the downstream ABA-responsive genes during water deficit stress, such as indeterminate domain 14 (IDD14) and nuclear factor Y subunit C (NF-YC) [28, 29]. However, although both WRKYs and ABFs are key TFs in ABA signaling, the mechanisms by which they cooperatively enhance drought tolerance remain less understood.

The plant cell cycle, a tightly orchestrated process driving development, relies on cyclins as phase-specific regulators [30]. D-type cyclins (CYCDs) are particularly vital for cell proliferation in different tissues, particularly in modulating the transition from postmitotic interphase to DNA synthesis phase [31]. The *Arabidopsis* genome contains 10 *D-type cyclin* (*CYCD*) genes, with *CYCD6;1* demonstrating specialized function in regulating root ground tissue patterning [32, 33]. Although these findings revealed the important role of *CYCD* genes in root development, whether WRKYs and *CYCD* genes could form a regulatory module in drought response remains unclear.

As a high-value perennial fruit crop, commercial *Citrus* seedlings are propagated by grafting and the rootstock significantly influences many horticultural traits, including drought tolerance [34, 35]. *Citrus reticulata* cv. Sanhu hongju (hereafter ‘Sanhu’), a native *Citrus* germplasm from Jiangxi Province, China, exhibits superior drought tolerance and serves as valuable rootstock material [36]. To analyze its transcriptional changes under drought, leaves of Sanhu after drought treatment were used for transcriptome sequencing, from which an ABA-responsive *CrWRKY57* was identified. Previously, WRKY57 was reported to confer elevation of ABA and drought tolerance by directly promoting the transcriptional levels of stress-responsive genes [37]. This study aimed to elucidate whether CrWRKY57 coordinates ABA-dependent drought responses through cell cycle regulation and to characterize its functional interplay with CrABF3.

## Results

### Identification and molecular characterization of *CrWRKY57*

Compared to the transcriptome data of Sanhu plants after withholding water for 1 d (DAW1, as control), there were 7519 differentially expressed genes (DEGs) in DAW4 and 6563 DEGs in DAW7. Cluster analysis of these DEGs is shown in Fig. S1A. The relative transcriptional expression levels of 14 selected DEGs were further analyzed using reverse transcription quantitative polymerase chain reaction (RT-qPCR). A strong correlation (*R*^2^ = 0.7156) between the results of RT-qPCR and RNA-seq, as indicated in Fig. S1B, confirmed the reliability of the RNA-seq data. From these DEGs, we selected one gene (Cs7g03080), which was putatively annotated as *CrWRKY57*, as a candidate transcription factor for further investigation.

**Figure 1:**
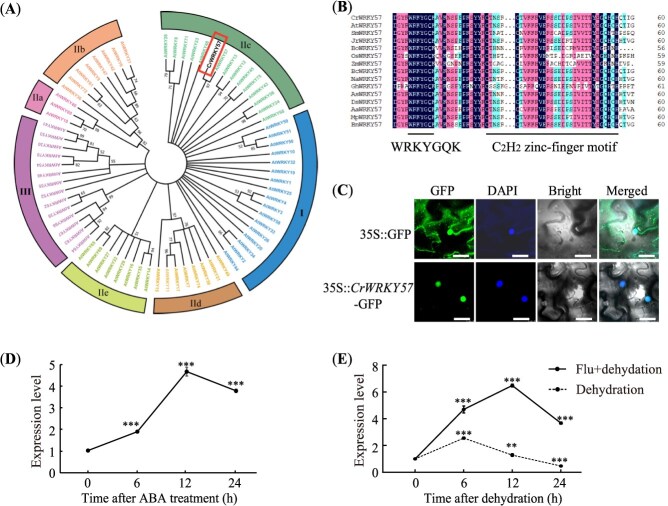
Protein characterization of CrWRKY57 and expression analysis. (**A**) Phylogenetic tree constructed using the sequence of CrWRKY57 (enclosed in a solid-line rectangle) and AtWRKY proteins from *Arabidopsis thaliana* (At). WRKY proteins are classified into three major groups (I, II, and III), with Group II further divided into five subgroups (IIa, IIb, IIc, IId, and IIe). (**B**) Alignment of the core amino acid sequences of CrWRKY57 with WRKY57 sequences from various species, including *Arabidopsis*, *S. miltiorrhiza* (Sm), *J. regia* (Jr), *B. olerace*a var. capitata (Bo), *O. sativa* (Os), *Z. mays* (Zm), *B. campestris* (Bc), *N. attenuata* (Na), *G. hirsutum* (Gh), *A. shenzhenica* (As), *D. catenatum* (Dc), *A. amnicola* (Aa), *M. pruriens* (Mp), and *B. napus* (Bn). The WRKYGQK heptapeptide and C_2_H_2_ zinc-finger motif were marked with straight lines. (**C**) Subcellular localization of CrWRKY57 in the nucleus, as indicated by GFP in *N. benthamiana* leaves. DAPI was used as a nuclear marker. Overlapping images are shown on the right. Scale bar = 20 μm. (**D**) Expression profiles of *CrWRKY57* under 100 μM ABA treatment. (**E**) Expression profiles of *CrWRKY57* under dehydration treatment and dehydration after being pre-incubated with 100 μM fluoridone (an ABA biosynthesis inhibitor) for 3 days. ^**^P < 0.01, ^***^P < 0.01, Student’s *t*-test.

The 876-bp open reading frame of the putative *CrWRKY57* was obtained by RT-PCR from Sanhu. It encodes a predicted protein of 291 amino acids, with a predicted molecular mass of 32.05 kDa and an isoelectric point of 6.19. A phylogenetic tree was constructed based on the obtained sequence of *WRKY57* and all the *Arabidopsis WRKY* members, in which *WRKY57* exhibited a closer relationship with *AtWRKY57* and both were clustered into Group IIc (Fig. 1A). Based on the alignment with its homologs from *Arabidopsis*, *Salvia miltiorrhiza*, *Juglans regia*, *Brassica olerace*a var. capitata, *Oryza sativa*, *Zea mays*, *Brassica campestris*, *Nicotiana attenuata*, *Gossypium hirsutum*, *Apostasia shenzhenica*, *Dendrobium catenatum*, *Anthurium amnicola*, *Mucuna pruriens*, and *Brassica napus*, the core amino acid region of our CrWRKY57 contained the conserved WRKYGQK heptapeptide and the typical C_2_H_2_ zinc-finger motif (Fig. 1B). Therefore, we named it *CrWRKY57*.

To determine its subcellular location, a construct driven by the CaMV 35S promoter was generated by fusing the *CrWRKY57* cDNA to the *yellow fluorescent protein* (*YFP*) gene. The fusion construct and the empty vector (EV) were transiently expressed in *Nicotiana benthamiana* leaves, respectively. Confocal microscopic observation showed the GFP signal from the EV was observed throughout the cells, but the GFP signal from the CrWRKY57-GFP fusion protein was detected exclusively in the nucleus and co-localized with the nuclear marker 4′,6-diamidino-2-phenylindole (DAPI), indicating that CrWRKY57 is a nuclear protein (Fig. 1C).

The expression profiles of *CrWRKY57* in response to ABA and dehydration were examined by RT-qPCR. In 2-month-old Sanhu plants, *CrWRKY57* transcripts progressively accumulated and peaked at 12 h post-treatment, showing more than a four-fold increase relative to the onset of exogenous ABA application (Fig. 1D). To determine whether the response of *CrWRKY57* to dehydration is dependent on ABA, 2-month-old seedlings were subjected to either direct dehydration or dehydration after pretreatment with fluridone (an inhibitor of ABA biosynthesis) for 3 days. Subsequent analysis of the transcriptional levels of *CrWRKY57* revealed that under direct dehydration treatment, *CrWRKY57* mRNA levels increased approximately 2.56-fold at 6 hours before declining thereafter (Fig. 1E). In contrast, in Sanhu seedlings pretreated with fluridone and then dehydrated, *CrWRKY57* expression was rapidly up-regulated, reaching 4.70-fold at 6 hours and peaking at 6.48-fold at 12 hours (Fig. 1E). These results suggest that *CrWRKY57* is inducible by both ABA and dehydration; however, the activation of *CrWRKY57* in response to dehydration appears to be independent of ABA.

### Overexpression and silencing of *CrWRKY57* in *Citrus* validate its role in drought tolerance

To further elucidate its role in drought tolerance, two *CrWRKY57*-overexpression lemon lines micrografted on *P. trifoliata* were obtained, designated as OE-1 and OE-2, and the untransformed micrografting plantlets (CK_L_) were used as control (Fig. S2). Meanwhile, RNA interference (RNAi) was used to knock down *CrWRKY57* in Sanhu, and its transcript abundance in nine lines was strongly repressed, from which RNAi-2 and RNAi-19 were randomly selected for further experiments, while the plantlets regenerated from untransformed Sanhu tissue were used for control (CKs) (Fig. S3).

**Figure 2:**
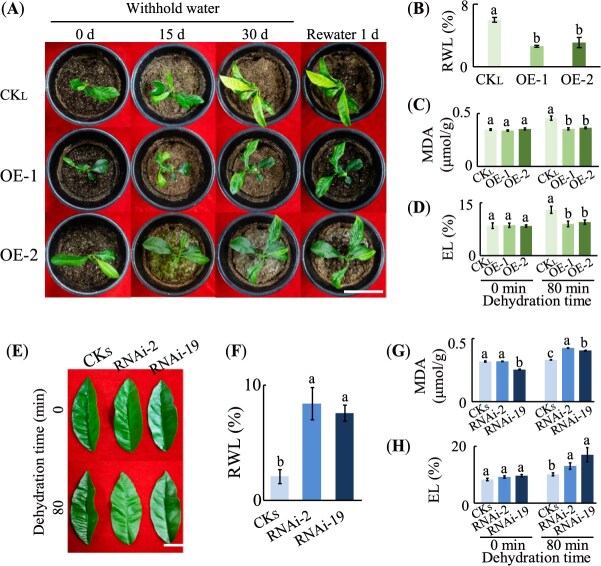
*CrWRKY57* positively regulates drought tolerance in *Citrus*. (**A**) Morphological comparison of potted control (CK_L_) and *CrWRKY57*-overexpression lemon lines (OE-1 and OE-2) before and after water withholding for 15 and 30 days, followed by 1 day of rehydration. Scale bar, 8 cm. (**B–D**) RWL (**B**), MDA content (**C**), EL (**D**) of detached leaves from CK_L_ and OE-1/OE-2 before and after 80 minutes of dehydration. (**E–H**) Morphology (**E**), RWL (**F**), MDA (**G**), and EL (**H**) of detached leaves from two *CrWRKY57*-RNA interference Sanhu lines (RNAi-2 and RNAi-19) and control (CKs) before and after 80 minutes of dehydration. Scale bar in (**E**), 3 cm, *n* = 3. Different letters marked above columns indicate statistically significant differences between samples at the same time point (*P* < 0.01).

**Figure 3:**
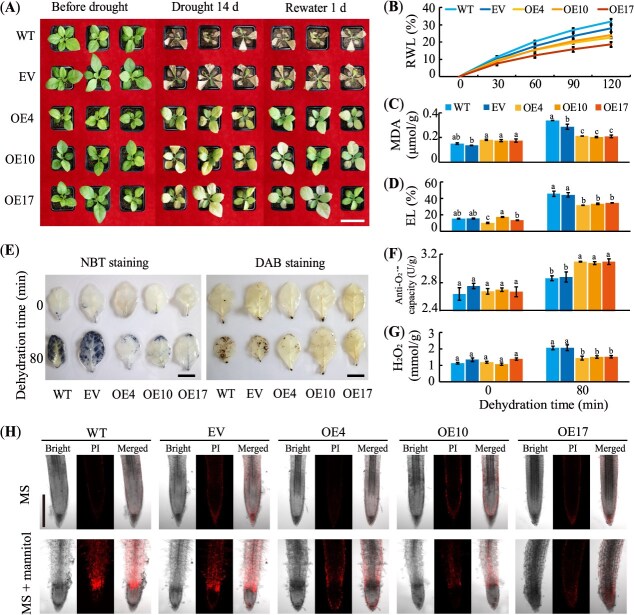
Overexpression of *CrWRKY57* enhances drought tolerance in tobacco (*N. tabacum*). (**A**) Phenotypes of wild-type (WT), tobacco transformed with an EV, and *CrWRKY57*-overexpression lines (OE4, OE10, and OE17) before and after drought treatment, followed by 1 day of rehydration. (**B**) RWL of detached leaves from WT, EV, OE4, OE10, and OE17 measured at 30-minute intervals. Scale bar, 7 cm. (**C–G)** MDA content (**C**), EL (**D**), *in situ* accumulation of O_2_^·–^ (left panel, revealed by histochemical staining with NBT) and H_2_O_2_ (right panel, revealed by DAB) (**E**), quantitative measurement of anti-O_2_^·–^ capacity (a negative parameter of O_2_^·–^ content) (**F**), H_2_O_2_ content (**G**) before and after 80 minutes of dehydration. Error bars indicate ±SE (*n* = 3). Bar in (**E**), 2 cm. Different letters marked above columns indicate statistically significant differences between samples at the same time point (*P* < 0.05). (**H**) Representative images of PI staining in tobacco root tip. Three-day-old seedlings germinated on an MS medium were transferred onto an MS medium with (upper panels) or without (lower panels) 0.4 M mannitol to grow for an additional 3 days before PI staining (*n* ≥ 50). Fluorescence indicates cell damage. Vertical bar, 500 μm. Different letters marked above columns indicate statistically significant differences between samples at the same time point (*P* < 0.01).

There was no obvious morphological difference between CK_L_ and OE-1 and OE-2 before and after withholding water for 15 days. However, after 30 days of water deprivation, CK_L_ suffered from more severe chlorosis compared to OE-1 and OE-2 (Fig. 3A). After resuming water for 1 day, OE-1 and OE-2 almost fully recovered from slight chlorosis, whereas CK_L_ showed no recovery (Fig. 2A). After 80 minutes of dehydration, relative water loss (RWL) in the control lemon lines was nearly double that of OE-1 and OE-2, while malondialdehyde (MDA) content and electrolyte leakage (EL) in the overexpression lines were significantly lower than those in CK_L_ (Fig. 2B–D, *P* < 0.01). Compared to CKs, the two transgenic Sanhu leaves (RNAi-2, RNAi-19) exhibited more severe wilting, along with significantly higher RWL, MDA content, and EL after 80 minutes of dehydration (Fig. 2E–H). Hence, these results collectively demonstrate that *CrWRKY57* plays a positive role in enhancing drought tolerance in *Citrus.*

### Heterologous overexpression of *CrWRKY57* confers drought tolerance in tobacco (*Nicotiana tabacum*)

To investigate its role in drought response, transgenic tobacco (*N. tabacum*) lines overexpressing *CrWRKY57* were generated (Fig. S4). Under normal growth conditions, there was no obvious morphological difference between the three OE lines and two controls. After 14 days of withholding water, wild-type (WT) and EV-transformed tobacco plants exhibited severe wilting, whereas three independent *CrWRKY57*-overexpression tobacco lines (OE4, OE10, OE17) maintained relatively better plant architectures. And they began to regenerate green leaves within 24 hours of rehydration (Fig. 3A). In dehydration assays, the RWL from detached leaves of the three OE tobacco lines was consistently lower than that of WT and EV plants at all measured time points (30, 60, 90, and 120 minutes) (Fig. 3B). Additionally, both MDA and EL levels in the OE lines were significantly reduced compared to WT and EV lines after 80 minutes of dehydration (Fig. 3C and D). The accumulation of superoxide radicals (O_2_^·–^) and hydrogen peroxide (H_2_O_2_) was qualitatively assessed using nitrotetrazolium blue chloride (NBT) and 3,3′-diaminobenzidine (DAB) staining, respectively. Dehydrated leaves of WT and EV plants exhibited deeper coloration, indicating higher levels of O_2_^·–^ and H_2_O_2_ compared to the OE lines (Fig. 3E). The quantitative measurements of anti-O_2_^.−^ capacity (a negative indicator of O_2_^·–^ level) and H_2_O_2_ concentration results were consistent with the histochemical staining images (Fig. 3F and G), suggesting that WT/EV accumulated more ROS than OE/4/10/17 did under dehydration.

To assess cellular damage, propidium iodide (PI), a dye that stains dead cells, was used to visualize root tissues. Red fluorescence, indicative of PI staining, was predominantly observed on the surfaces of root tips in all 6-day-old tobacco seedlings grown on a Murashige and Skoog (MS) medium (Fig. 3H, upper panel). However, when exogenous mannitol was applied to simulate dehydration, the fluorescence intensity was markedly stronger in the root tips of WT and EV lines compared to those of the OE lines grown on an MS medium supplemented with mannitol (Fig. 3H, lower panel), suggesting increased cell damage in WT and EV plants.

### *CrWRKY57* regulates root development

**Figure 4:**
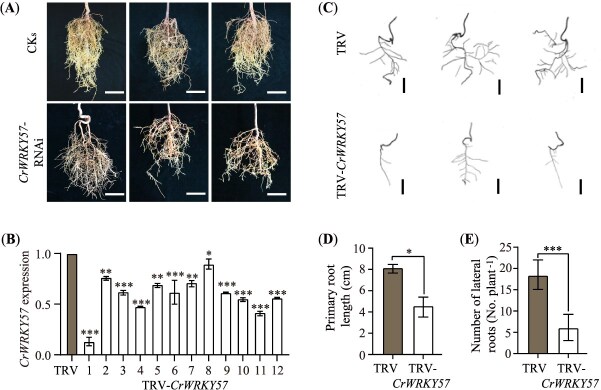
*CrWRKY57* regulates root growth in *Citrus*. (**A**) Representative root morphology of 2-year-old *CrWRKY57*-RNAi plants and their controls (CKs). Scale bars, 5 cm. (**B**) RT-qPCR analysis of the *CrWRKY57* expression level between control (TRV, seedlings carrying the empty TRV vectors) and 12 *CrWRKY57*-VIGS Sanhu lines (TRV-*CrWRKY57*). (**C**) Representative scanning images of roots from TRV-*CrWRKY57* and TRV plants. Scale bars, 1 cm. (**D**, **E**) Average primary root length (**D**) and lateral root number (**E**) of TRV-*CrWRKY57* and TRV plants. Error bars indicate ±SE (*n* = 12). ^*^P < 0.05, ^**^P < 0.01, ^***^P < 0.01, Student’s *t*-test.

Compared to the 2-year-old *CrWRKY57*-RNAi plants, we observed that the control Sanhu plants had a whiter root system with greater volume (Fig. 4A). To further investigate its role in root development, *CrWRKY57* was silenced in Sanhu plants using the virus-induced gene silencing (VIGS) system. Eleven seedlings exhibiting a significant reduction in *CrWRKY57* expression were selected as VIGS lines and referred to as TRV-*CrWRKY57* (Fig. 4B). Based on the scanning images, the control lines (TRV) carrying the EVs (pTRV1 + pTRV2) exhibited obviously longer primary root length and a greater number of lateral roots compared to the TRV-*CrWRKY57* plants (Fig. 4C). Indeed, the average primary root lengths of the TRV control and TRV-*CrWRKY57* were 8.14 and 4.56 cm, respectively, and the average lateral root numbers were 18 and 6 per plant, respectively (Fig. 4D and E).

Roots were also compared in *CrWRKY57*-overexpression tobacco (*N. tabacum*) and control seedlings. Since OE tobacco lines germinated later than controls (Fig. S5A), all seedlings were transferred to new Petri dishes to monitor root growth after germination on an MS medium for approximately 3 days. Seventeen days after germination (DAG), the roots of OE lines were significantly longer compared to those of WT and EV controls (Fig. S5B, upper panel). On 23 DAG and 30 DAG, the roots of WT and EV controls had nearly ceased elongation, whereas the roots of OE lines continued to grow (Fig. S5B, middle and lower panels). When the 4-day-old tobacco seedlings were transferred to an MS medium supplemented with 0.4 M mannitol to mimic dehydration, all lines exhibited retarded growth and reduced lateral root formation (Fig. S5C). However, the primary roots of OE10 and OE17 were notably longer than those of controls (Fig. S5C). On an MS medium supplemented with fluoridone (an ABA biosynthesis inhibitor), all tobacco lines showed increased lateral root formation, but OE lines still demonstrated longer primary roots overall (Fig. S5D). Collectively, these findings suggest that *CrWRKY57* promotes primary root elongation under both mannitol-induced dehydration and ABA biosynthesis inhibition.

### CrWRKY57 positively regulates ABA signaling and physically interacts with CrABF3

**Figure 5:**
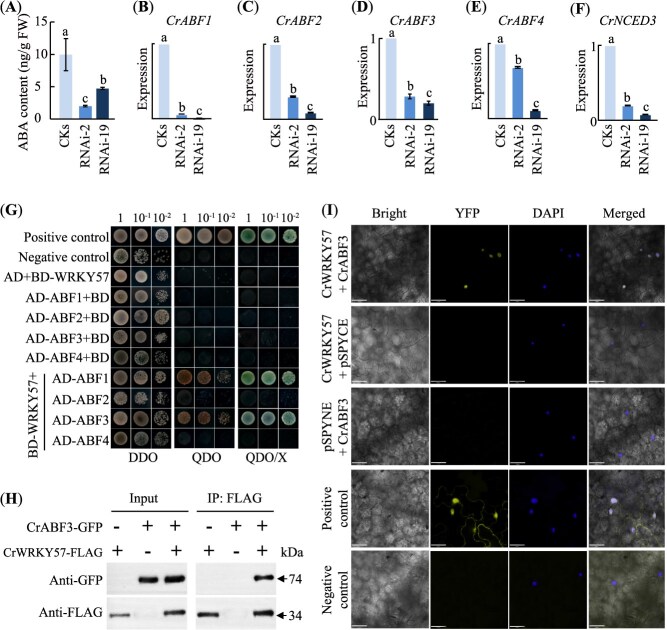
CrWRKY57 affects ABA biosynthesis and interacts with CrABF3. (**A**) ABA content in *CrWRKY57*-RNA interference Sanhu lines (RNAi-2 and RNAi-19) and control (CKs). (**B–F**) RT-qPCR analysis of *CrABF1* (**B**), *CrABF2* (**C**), *CrABF3* (**D**), *CrABF4* (**E**), and *CrNCED3* (**F**) in RNAi lines and CKs. Values are means ± SE (*n* = 3). Different letters above columns indicate significant differences (*P* < 0.05). (**G**) Y2H assay. *CrABFs* were fused with the activation domain (AD), and *CrWRKY57* was fused with the binding domain (BD). Yeast cells were grown on a control medium (SD/−Leu/−Trp, DDO) and selective media (SD/−Leu/−Trp/−Ade/-His, QDO; QDO added with X-α-gal, QDO/X). Positive control, pGBKT7–53 + pGADT7-T. Negative control, pGBKT7-Lam + pGADT7-T. (**H**) *In vivo* Co-IP assay of CrWRKY57 and CrABF3. Input shows expression levels of GFP-tagged CrABF3 (CrABF3-GFP) and FLAG-tagged CrWRKY57 (CrWRKY57-FLAG) in total protein extracts. Immunoprecipitated proteins (IP: FLAG) were analyzed by immunoblotting with anti-GFP or anti-FLAG antibodies. (**I**) BiFC assay using co-expression of pSPYNE-35S-*CrWRKY57* (CrWRKY57-nYFP) + pSPYCE-35S-*CrABF3* (CrABF3-cYFP), pSPYNE-35S-53 + pSPYCE-35S-T (positive control), and pSPYNE-35S + pSPYCE-35S (negative control) in *N. benthamiana* leaves. Scale bars, 32 μm.

The delayed germination phenotype in CrWRKY57-overexpression *N. tabacum* lines germinated later (Fig. S5A) prompted us to investigate its potential role in ABA biosynthesis. As expected, ABA levels in CKs (9.96 ng/g) were significantly higher than that in RNAi-2 (2.02 ng/g) and RNAi-19 (4.74 ng/g) (Fig. 5A, *P* < 0.05). Meanwhile, we also observed a significant reduction in the expression of *ABF1–4* and *9-cis-epoxycarotenoid dioxygenase* (*NCED3*), which encodes the rate-limiting enzyme in ABA biosynthesis (Fig. 5B–F, *P* < 0.05). Conversely, *CrWRKY57*-overexpression lemon samples showed about 1.4-fold increase in ABA and significantly elevated transcript levels of *ABF1*, *ABF3*, *ABF4*, and *NCED3* (Fig. S6, *P* < 0.05). Subsequently, we analyzed the promoter regions of *CrABF1*–*4* and found that the promoters of *CrABF1* and *CrABF3* both contain one W-box motif (Fig. S7). The results of yeast one-hybrid (Y1H) and dual luciferase (LUC) assays demonstrated that CrWRKY57 binds to the *CrABF1* promoter and suppresses its expression, but no direct regulatory effect on *CrABF3* was observed (Fig. S8). Despite the inconsistent result of *CrABF1*, these findings suggest that overall CrWRKY57 positively influences ABA signaling.

In *Arabidopsis*, IDD14 could interact with all the four ABFs to cooperatively increase drought tolerance [28]. Therefore, we testified whether CrWRKY57 and CrABFs could interact at the protein level. To this end, the protein–protein interactions between CrWRKY57 and CrABFs were first tested using the yeast two-hybrid (Y2H) system, and the results revealed that CrWRKY57 could interact with CrABF1 and CrABF3, but not with CrABF2 and CrABF4 (Fig. 5G). Unlike *CrABF2–4*, *CrABF1* was significantly downregulated by dehydration (Fig. S9). Therefore, CrABF3 was chosen for further investigation to validate its physical interaction with CrWRKY57. The co-immunoprecipitation (Co-IP) results showed clear bands for both FLAG-tagged CrWRKY57 and GFP-tagged CrABF3 in the input and immunoprecipitated samples, indicating a successful *in vivo* interaction between the two proteins (Fig. 5H). Additionally, bimolecular fluorescence complementation (BiFC) in tobacco (*N. benthamiana*) leaves demonstrated strong nuclear fluorescence upon co-expression of CrWRKY57 and CrABF3, confirming their interaction within the nucleus (Fig. 5I).

### CrWRKY57 and CrABF3 bind to the promoter of *CrCYCD6;1* and activate its expression

**Figure 6:**
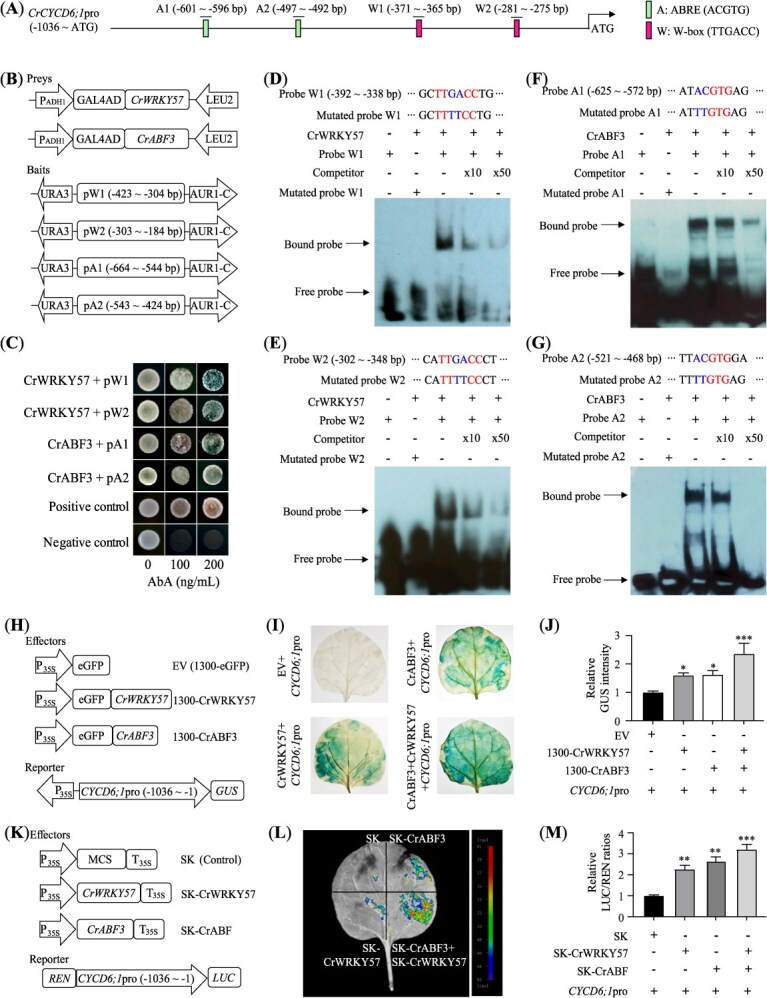
CrWRKY57 and CrABF3 co-activate *CrCYCD6;1*. (**A**) Schematic diagram of the partial promoter region of *CrCYCD6;1*, showing W-box (W1 and W2) and ABRE *cis*-elements (A1 and A2). (**B**) Prey (CrWRKY57 and CrABF3) and bait (pW1, pW2, pA1, and pA2 containing W1, W2, A1, and A2, respectively) constructs used for Y1H analysis. (**C**) Growth of yeast cells co-transformed with prey and bait on an SD/-Ura/−Leu medium added with 0, 100, and 200 ng/mL AbA. Positive control, pAbAi-p53 and pGADT7-p53; negative control, baits + pGADT7. (**D–G**) EMSA assay showing specific binding of CrWRKY57 to W1 (**D**) and W2 (**E**), and CrABF to A1 (**F**) and A2 (**G**) of the *CrCYCD6;1* promoter. The purified His-CrWRKY57 and His-CrABF3 proteins are incubated with biotin-labeled wild-type or mutated probes, with or without unlabeled competitor probes. (**H**) Schematic diagram of the effectors and reporter for GUS staining. EV, pCAMBIA1300-eGFP EV; 1300-CrWRKY57/CrABF3, pCAMBIA1300-eGFP ligated with CrWRKY57/CrABF3. (**I**, **J**) Representative images (**I**) and quantification (**J**) of transient GUS expression analysis using EV, 1300-CrABF3, 1300-CrWRKY57, and 1300-CrWRKY57 + 1300-CrABF3 as effectors and *CrCYCD6;1*pro:GUS as the reporter. GUS activity of the control (*N. benthamiana* leaves co-transformed with an empty effector vector and reporter) was taken as 1 for normalization. (**K**) Schematic diagram of effectors and reporter for dual luciferase assay (LUC). SK, empty pGreen II 62-SK; SK-CrWRKY57/CrABF3, pGreen II 62-SK ligated with CrWRKY57/CrABF3. pGreen II 0800-LUC ligated with the *CrCYCD6;1* promoter fragment was used as the reporter. The LUC/REN ratio of SK and reporter is set as 1 for normalization. (**L**, **M**) Representative bioluminescence image (**L**) and quantitative analysis (**M**) of the capability of CrWRKY57 and CrABF3 to activate *CrCYCD6;1*pro-LUC activity in *N. benthamiana* leaves. +, presence; −, absence. Error bars indicate ±SE (*n* = 3). ^*^P < 0.05, ^**^P < 0.01, ^***^P < 0.01, Student’s *t*-test.

*CYCD6;1* plays a critical role in root ground tissue patterning [32, 33]. Notably, *CYCD6;1* expression was elevated in *CrWRKY57*-overexpressing lemon plants but reduced in *CrWRKY57*-RNAi Sanhu lines (Fig. S10). To investigate whether CrWRKY57 regulates *CrCYCD6;1* expression, we obtained a 1036-bp sequence upstream of the ATG start codon from the *CrCYCD6;1* promoter. Surprisingly, this region contained two W-box elements (W1 and W2) and two ABRE elements (A1 and A2), suggesting that *CrCYCD6;1* could be regulated by both CrWRKY57 and CrABF3 (Fig. 6A). To explore this potential regulation, two prey (CrWRKY57 and CrABF3) and four bait (pW1, pW2, pA1, pA2) vectors were constructed for Y1H assay (Fig. 6B). The Y1H results demonstrated that yeast cells co-transformed with each prey–bait combination, as well as positive controls, grew robustly on SD/-Ura/−Leu media supplemented with Aureobasidin A (AbA), indicating that CrWRKY57 and CrABF3 can bind to the W-box and ABRE elements within the *CrCYCD6;1* promoter (Fig. 6C). To further confirm these interactions, we performed electrophoretic mobility shift assays (EMSAs) using His-tagged fusion proteins of CrWRKY57 and CrABF3. Incubation of these fusion proteins with labeled probe containing the native *cis*-elements resulted in observable shifts due to protein–DNA complex formation. The addition of unlabeled competitor probes diminished this binding, while mutated probes failed to produce any shift when incubated with the fusion proteins (Fig. 6D–G). These findings confirmed that CrWRKY57 and CrABF3 directly and specifically interact with the *CrCYCD6;1* promoter through its W-box and ABRE elements, respectively.

To determine whether CrWRKY57 and CrABF3 can activate the *CrCYCD6;1* promoter through their respective binding sites (W1/2 for CrWRKY57 and A1/2 for CrABF3), we utilized the 1036-bp promoter region of *CrCYCD6;1* in β-glucuronidase (GUS) staining and dual luciferase (LUC) assays. For the GUS staining, a transient expression assay was conducted by co-expressing the effector proteins together with the GUS reporter gene, driven by the *CrCYCD6;1* promoter, in the leaves of *N. benthamiana* (Fig. 6H and I). Histochemical staining revealed that when an EV (pCAMBIA1300-eGFP, EV) was co-expressed with the reporter, no GUS activity was observed. However, both 1300-CrWRKY57 and 1300-CrABF3 individually activated GUS expression driven by the *CrCYCD6;1* promoter. Notably, the combination of 1300-CrWRKY57 + 1300-CrABF3 significantly intensified GUS expression (Fig. 6I and J). Similarly, in the dual LUC assay, visualization of LUC fluorescence and quantitative measurement of the LUC/REN ratios confirmed that both CrWRKY57 and CrABF3 could significantly enhance the transcriptional activity of the *CrCYCD6;1* promoter. Moreover, the activation was more pronounced when CrWRKY57 and CrABF3 acted together, indicating a synergistic effect between these two TFs (Fig. 6K–M).

### Silencing *ABF3* reduces drought tolerance and root development in *Citrus*

**Figure 7:**
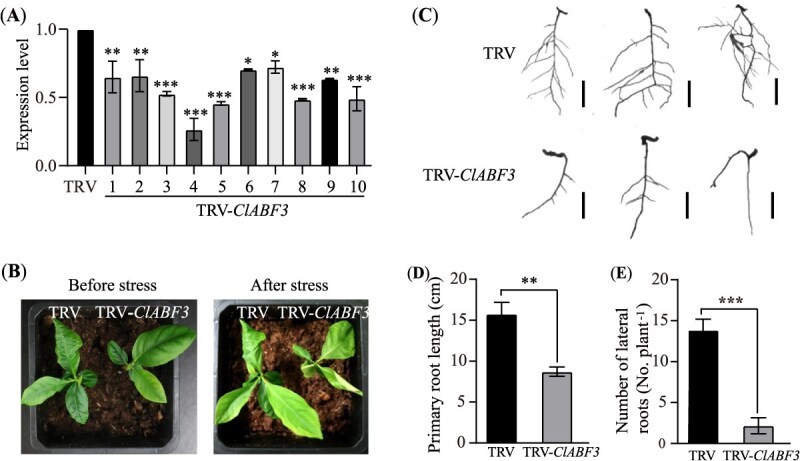
*ClABF3* regulates drought tolerance and root development. (**A**) RT-qPCR analysis of the *ClABF3* expression level between control (TRV, seedlings carrying the empty TRV vectors) and *ClABF3*-VIGS Sanhu lines (TRV-*ClABF3*). (**B**) Representative morphology of 40-day-old TRV-*ClABF3* and TRV control plants before and after a 10-day drought treatment. (**C–E**) Representative root images (**C**), primary root length (**D**), and number of lateral roots per plant (**E**) in TRV-*ClABF3* and TRV seedlings. Scale bars in (**C**), 2 cm. ^*^P < 0.05, ^**^P < 0.01, ^***^P < 0.001, Student’s *t*-test.

Due to the limited availability of Sanhu seeds, we employed VIGS to silence *ClABF3* (a homolog of *CrABF3*, sharing 97% similarity at the protein level) to investigate its role in drought tolerance and root development in *Citrus limon*. The VIGS lines (TRV-*ClABF3*) showed 25%–75% reduction in *ClABF3* expression compared to their TRV control plants (Fig. 7A). After withholding water for 10 days, TRV-*ClABF3* plants exhibited severe wilting and leaf curling, indicating increased drought sensitivity (Fig. 7B). Additionally, the average primary root length of TRV-*ClABF3* seedlings was about 1.9 times shorter than that of the control (TRV) seedlings (Fig. 7C and D). Furthermore, TRV-*ClABF3* seedlings exhibited a 6.5-fold reduction in average lateral root number per plant compared to the control (Fig. 7C and E). These findings suggest the positive role of *ABF3* in drought tolerance and root development in *Citrus*.

### Silencing of *CrCYCD6;1* inhibits root development

**Figure 8:**
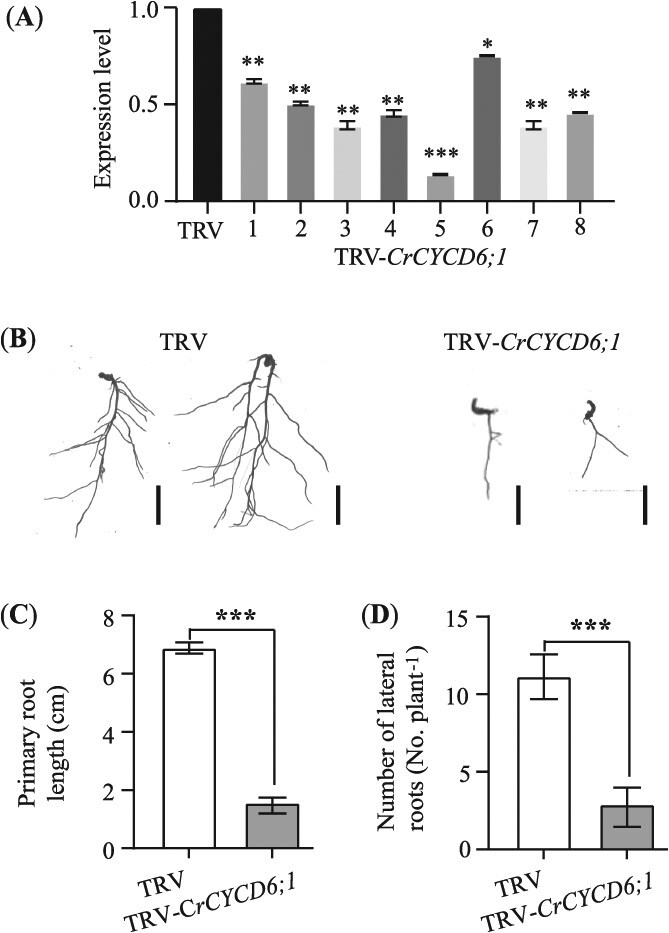
*CrCYCD6;1* participates in root development. (**A**) RT-qPCR analysis of the *CrCYCD6;1* expression level between control (TRV, seedlings carrying the empty TRV vectors) and eight *CrCYCD6;1*-VIGS Sanhu lines (TRV-*CrCYCD6;1*). (**B–D**) Representative root images (**B**), primary root length (**C**), and number of lateral roots per plant (**D**) in TRV-*CrCYCD6;1* and TRV seedlings. Scale bars in (**B**), 1 cm. ^*^P < 0.05, ^**^P < 0.01, ^***^P < 0.001, Student’s *t*-test.

Although the work of Xie *et al.* has implicated the role of *CYCD6;1* in root development [33], we further validated its function by silencing *CrCYCD6;1* using the VIGS system in the Sanhu background. Eight independent TRV-*CrCYCD6;1* lines with 20%–75% reduction in *CrCYCD6;1* expression, relative to the EV control (TRV), were selected for analysis (Fig. 8A). The average primary root length of TRV-*CrCYCD6;1* seedlings was 1.55 cm, significantly shorter than that of the control plants, 6.89 cm (Fig. 8B and C*, P* < 0.001). Furthermore, TRV-*CrCYCD6;1* seedlings exhibited a 3.9-fold reduction in the average lateral root number per plant relative to controls (Fig. 8B and D, *P* < 0.001). These results indicate that *CrCYCD6;1* is crucial for normal root growth and development processes, contributing to both primary and lateral root formation.

## Discussion

WRKY TFs act as key regulators of drought tolerance, participating in various physiological and developmental processes [5, 11, 38–41]. In *Arabidopsis*, WRKY57 directly enhances the expression levels of genes encoding dehydration-responsive element 29A (RD29A) and 9-*cis*-epoxycarotenoid dioxygenase (NCED3, the rate-limiting enzyme in ABA biosynthesis), thereby increasing ABA levels and conferring drought tolerance [37]. Heterologous overexpression of *AtWRKY57* enhanced drought tolerance in *O. sativa* [42]. However, the biological role of *WRKY57* and its specific mechanisms in drought tolerance remain unclear. In this study, we identified a drought-responsive and ABA-inducible Group IIc WRKY from *C. reticulata* cv. Sanhu hongju, designated as *CrWRKY57*. Consistent with previous findings, CrWRKY57 acts as a positive regulator of drought tolerance. We further demonstrated that CrWRKY57 positively participates in ABA signaling through its physical interaction with CrABF3. The CrWRKY57–CrABF3 heterodimeric complex induces the expression of the root development-related gene *CrCYCD6;1*, promoting root elongation and enhancing drought tolerance. Collectively, our findings reveal a previously unidentified role of WRKY57 in ABA signaling and drought tolerance.

### CrWRKY57 interacts with CrABF3 as a positive regulator in drought response and ABA signaling

WRKY TFs can act as either positive or negative regulators in ABA-mediated drought tolerance. PoWRKY71 from *Paeonia ostii* enhances drought tolerance by activating the expression of the gene encoding the photosynthesis-related light-harvesting chlorophyll a/b-binding 151, thereby stabilizing photosynthesis under drought stress [5]. In *Populus tomentosa*, allelic variations in the *PtoWRKY68* modulate ABA signaling and accumulation [43]. In contrast, some WRKYs, such as WRKY40, WRKY18, and WRKY60, negatively regulate ABA signaling by repressing the expression of *ABF4*, *ABA-insensitive 4* (*ABI4*), *ABI5*, and *MYB* [44]. OsWRKY5 negatively regulates ABA-induced drought tolerance in rice by inhibiting the expression of *OsMYB2* [40]. In *Solanum lycopersicum*, *WRKY81* also acts as a negative regulator for drought tolerance by influencing stomatal closure [38]. Here, we found that overexpression of *CrWRKY57* improved drought tolerance in both *C. limon* and *N. tabacum*, whereas *CrWRKY57*-RNAi lines of Sanhu showed increased susceptibility to dehydration compared to controls, indicating that CrWRKY57 acts as a positive regulator of drought tolerance.

WRKYs can interact with various factors to fine-tune the transcriptional regulatory networks involved in stress responses [10, 11]. In the Chinese wild grape *Vitis quinquangularis*, Vqwrky53 interacts with VqMYB14 and VqMYB15 to promote stilbene synthesis and disease resistance [45]. PoWRKY69 enhances drought response by interacting with valine-glutamine 11 (PoVQ11) to regulate fructose accumulation [5]. Here, although Y1H and LUC assays demonstrated CrWRKY57 directly binds to the *CrABF1* promoter and inhibits its transcription, *CrWRKY57*-RNAi Sanhu plants exhibited reduced expression levels of *CrABF1–4* and *CrNCED3*, along with decreased ABA content. Conversely, those genes’ expression levels were upregulated in *CrWRKY57*-overexpression lemon plants. These findings suggest that CrWRKY57 positively regulates the ABA signaling pathway, not by directly upregulating *CrABFs*. Instead, our subsequent Y2H, Co-IP, and EMSA results demonstrated that CrWRKY57 directly interacts with CrABF3 at the protein level, confirming its role as a positive regulator in the ABA signaling pathway.

### WRKY57 acts as a positive regulator in root development

Root architecture plays a fundamental role in plant survival and productivity [46, 47]. Longer roots enhance water uptake from deeper soil layers, and architectural remodeling is critical for drought adaptation [3, 48]. While ABA promotes stomatal closure to reduce water loss, it simultaneously restricts root system expansion through inhibition of quiescent center activity in *Arabidopsis*, thereby suppressing both primary root elongation and lateral root initiation [49, 50]. Exogenous ABA also inhibits primary root elongation by promoting auxin biosynthesis and repressing the transcription of a B-type cyclin, *CYCB1;1*, [51, 52]. In this study, overexpressing *CrWRKY57* in lemon significantly increased ABA levels, while silencing *CrWRKY57* in Sanhu via VIGS reduced primary root length and lateral root numbers as well as ABA levels. On one hand, these results suggest that the *CrWRKY57*-mediated increase in ABA might not have an inhibitory effect on primary root development. On the other hand, they also raise the possibility that *CrWRKY57* could be fundamental to primary and lateral root development. Indeed, *CrWRKY57*-overexpression *N. tabacum* exhibited prolonged primary root elongation on an MS medium, continuing growth until 30 days after germination (DAG), compared to controls (WT and EV) that ceased elongation at 23 DAG. Additionally, under mannitol or fluoridone (an inhibitor of ABA biosynthesis) treatments, *CrWRKY57*-overexpression *N. tabacum* showed faster primary root elongation than the controls. Interestingly, under mannitol-induced dehydration conditions, while lateral root formation was severely suppressed in *CrWRKY57*-overexpression tobacco lines, primary root elongation in these lines was faster than in controls. Therefore, we propose that CrWRKY57 functions as a positive regulator in promoting primary root growth under drought conditions. However, it cannot counteract the drought-induced inhibition of lateral root development.

### CrABF3 positively regulates drought response and root growth

Among the four ABFs, *AREB1*/*ABF2*, *AREB2*/*ABF4*, and *ABF3* have largely overlapping functions in drought tolerance, serving as master regulators of ABA signaling and drought responses [53]. Unlike *ABF3* or *ABF4*, the overexpression of the intact *ABF2* gene does not significantly enhance drought tolerance in *Arabidopsis* [18, 28]. However, overexpression of *PtrABF2* from *P. trifoliata* significantly enhances drought tolerance in *C. limon* [26]. *PtrABF4* also positively regulates dehydration tolerance by reducing ROS accumulation and stomatal density [8]. Although the role of *ABF3* in *Citrus* and its relatives has not been reported previously, ABF3 has been shown to enhance drought tolerance in other species, such as *Populus euphratica* and *Glycine max* [7, 54]. Our results demonstrate that silencing *ClABF3* in *C. limon* significantly reduces drought tolerance and impairs root development, as evidenced by shorter primary roots and fewer lateral roots in TRV-*ClABF3* seedlings. These effects may be attributed to the disruption of ABA signaling, which plays a critical role in regulating root architecture and elongation, as well as integrating environmental cues with developmental processes. *ABF3* appears to be a key player in this integration [55]. These findings contribute to the growing body of evidence emphasizing the importance of ABF TFs in plant stress responses and developmental regulation.

**Figure 9:**
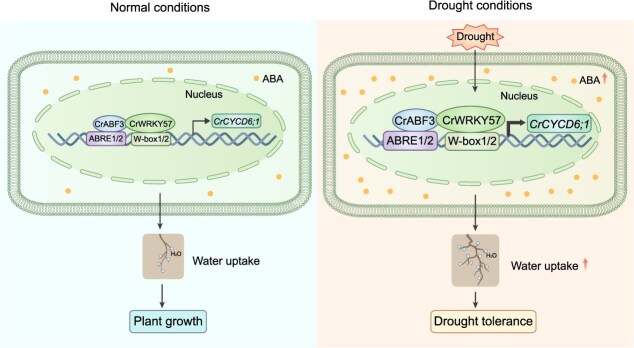
A proposed model of CrWRKY57-mediated drought tolerance in *Citrus*. CrWRKY57 and CrABF3 co-activate the transcriptional expression of *CrCYCD6;1*, regulating root development and increasing drought tolerance.

### WRKY57 and ABF3 cooperatively participated in root development via regulating *CrCYCD6;1*

D-type cyclins (CYCDs) are crucial cell cycle activators in the root apex to promote root emergence [56]. As the two master regulators of root growth and development, SHORT-ROOT (SHR) and SCARECROW (SCR) control cell cycle progression via the synergistic activation of *CYCD6;1* [32, 57, 58]. Our results demonstrate that CrWRKY57 and its co-activator CrABF3 positively regulate the transcript accumulation of *CrCYCD6;1* and directly activate its transcription. And as expected, silencing *CrWRKY57, CrABF3*, and *CrCYCD6;1* via the VIGS system all severely impaired the development of both primary and lateral roots in *Citrus*. These findings collectively suggest that CrWRKY57 and CrABF3 likely regulate *CrCYCD6;1*-mediated promotion of primary root elongation and lateral root emergence under normal conditions. However, considering the inhibition of lateral root emergence in *CrWRKY57*-overexpression *N. tabacum* grown on an MS medium supplemented with mannitol, we propose that this mechanism may prioritize primary root growth over lateral root formation under drought conditions, as longer roots with reduced branching angles could access deeper water sources [3]. It is well established that lateral roots are primarily promoted by auxin [59]. Notably, ABI3 (another ABA-responsive TF) integrates ABA and auxin signaling to regulate primary root growth during dehydration [6]. Jiang *et al.* [37] reported that WRKY57 functions as a key integrator between the jasmonate and auxin signaling pathways to coordinately regulate leaf senescence in *Arabidopsis*. Therefore, it would be interesting in future studies to explore whether CrWRKY57 participates in diverse hormone signaling pathways to coordinate root development and drought responses.

### Conclusion

Taken together, we propose a working model for the CrWRKY57-CrABF3-*CrCYCD6;1* module in *Citrus*: drought induces CrWRKY57 and CrABF3, which respectively bind to the *cis*-acting elements in the promoter region of *CrCYCD6;1* and upregulate its expression. Additionally, the physical interaction between CrWRKY57 and CrABF3 enhances this activation effect, leading to increased ABA levels and modified root morphological features that can improve water uptake and consequently improve drought tolerance (Fig. 9). Our findings reveal a novel regulatory mechanism of root development in *Citrus* under drought stress and would provide potential targets for improving drought resilience in *Citrus* rootstock through genetic engineering.

## Materials and methods

### Plant materials and treatments

To investigate the responses of *C. reticulata* (Sanhu) to various treatments, 1-year-old Sanhu plants cultivated in the greenhouse at Gannan Normal University were watered continuously for 3 days and then subjected to water withholding for 1, 4, and 7 days (DAW1, DAW4, DAW7). Leaf samples collected at each time point were used for RNA-seq analysis. For dehydration treatment, 2-month-old uprooted Sanhu plants were placed on clean filter paper at room temperature, and leaves were sampled at 0, 6, 12, and 24 hours. For exogenous application of ABA and fluoridone (an ABA biosynthesis inhibitor), 2-month-old Sanhu plants were treated with 100 μmol/L solutions of ABA or fluoridone, respectively, and leaf samples were collected at 0, 6, 12, and 24 hours post-treatment.

After germination on an MS medium, T2 *CrWRKY57*-overexpression tobacco (*N. tabacum*) lines with at least two true leaves, and the control plants were transplanted into pots containing a 3:1 mixture of nutritive soil and vermiculite. The plants were grown in an incubator (25°C; 16-hour light/8-hour dark) for 1 month under well-watered conditions. Subsequently, the tobacco plants were subjected to natural drought treatment for 14 days, followed by resuming water to recover.

For dehydration treatment of *CrWRKY57*-overexpression *N. tabacum* and lemon (*C. limon*), *CrWRKY57*-RNAi Sanhu, and their corresponding controls, mature leaves were detached and placed on clean filter papers at room temperature and collected at designated time points. For drought treatment of the *CrWRKY57*-RNAi Sanhu and control (CKs), 6-month-old plants growing in soil pots (height: 23 cm, diameter: 16.5 cm) were watered for 3 days and then withheld water for 15 and 30 days, followed by normal irrigation.

### RNA-seq analysis

Tender Sanhu leaves were used for total RNA extraction using RNAiso Plus reagent (TaKaRa, Beijing, China), followed by DNase I treatment to eliminate genomic DNA contamination. After first-strand cDNA synthesis was performed using the RevertAid First Strand cDNA Synthesis Kit (Thermo Fisher Scientific, Waltham, MA, USA), cDNA library construction and RNA-seq were conducted on an Illumina HiSeq™ 2000 platform by Novogene Co. Ltd (Beijing, China). The raw sequencing data have been deposited in the National Center for Biotechnology Information (NCBI) under BioProject accession PRJNA1007268.

We aligned sequencing reads to *C. sinensis* reference genome using Bowtie and Tophat with default parameters [60, 61]. Gene expression levels were quantified and normalized as fragments per kilobase of exon per million fragments mapped values following the methodology of Trapnell *et al.* [62]. DEGs were identified as genes with a corrected *P* value <0.05 and an absolute value of log_2_(Sample1/Sample2) >1. Goseq was used for gene ontology enrichment analysis [63]. Pathway mapping of DEGs and TFs identification were conducted through the Kyoto Encyclopedia of Genes and Genomes (KEGG) Orthology Based Annotation System (KOBAS) and iTAK, respectively [64, 65].

### Sequence isolation and promoter cloning

The full-length coding sequences (CDSs) and promoter regions of genes were isolated from genomic DNA and cDNA of Sanhu leaves, respectively, by gene-specific primers designed according to homologous sequences of sweet orange (*C. sinensis*). Sequences of *Arabidopsis* WRKY family and WRKY57 homologous sequences from *S. miltiorrhiza*, *J. regia*, *B. olerace*a var. capitata, *O. sativa*, *Z. mays*, *B. campestris*, *N. attenuata*, *G. hirsutum*, *A. shenzhenica*, *D. catenatum*, *A. amnicola*, *M. pruriens*, and *B. napus* were downloaded from Phytozome v12.1 (https://phytozome.jgi.doe.gov/pz/portal.html). Phylogenetic analysis and the core amino acid sequence alignment of WRKYs were performed with MAGE7.0 and DNAMAN 8.0, respectively. Putative *cis*-elements were predicted using PLACE (http://www.dna.affrc.go.jp/PLACE/).

### Quantitative RT-qPCR analysis

Following RNA extraction and cDNA synthesis as previously described, RT-qPCR was conducted using FastStart Essential DNA Green Master on a LightCycler^®^ 96 system (Roche, Basel, Switzerland) according to the manufacturer’s instructions. The relative expression was calculated based on the 2^–ΔΔCt^ method [66], with normalization to the *Actin* reference gene. Each reaction was performed three times.

### Subcellular localization analysis

The full-length cDNA of *CrWRKY57* (termination codon excluded) was ligated to the pBI121-GFP vector containing the green fluorescent protein (GFP), generating the *35S::CrWRKY57*-*GFP* construct. Both recombinant and control vector (*35S::GFP*) plasmids were transformed into 50-day-old *N. benthamiana* leaves via *Agrobacterium tumefaciens*-mediated transformation [67]. Nuclei were stained with DAPI. Fluorescence signals were captured using the TCS SP8 confocal microscope system (Leica Microsystems, Germany).

### Generation and identification of transgenic plants

The *p35S::WRKY57* construct was generated by inserting the *CrWRKY57* coding region between the XbaI and SmaI sites of the pBI121 vector. RNA interference (RNAi) vectors were created by introducing a *CrWRKY57* fragment into the pHELLSGATE2 vector through BP recombination reaction (Invitrogen, Japan). The overexpression and RNAi vectors were mobilized into *A. tumefaciens* strain GV3101.

Epicotyls from sterilized lemon or Sanhu seeds were used to generate *CrWRKY57*-overexpression lemon (*C. limon*) and *CrWRKY57*-RNAi Sanhu plants [67]. Briefly, sterilized seeds were placed in the MT medium and kept in darkness for 20 days. Subsequently, the obtained epicotyls were cut into 0.5-cm fragments and incubated with *A. tumefaciens* solution containing the *CrWRKY57*-overexpression vector or *CrWRKY57*-RNAi vector for 15 minutes. The regenerated plantlets from the fragments were verified by regular PCR and RT-qPCR. The positive *CrWRKY57*-RNAi Sanhu lines regenerated roots easily and were vegetatively multiplied, whereas *CrWRKY57*-OE lemon lines were difficult to regenerate roots and grafted on etiolated *P. trifoliata* rootstocks for propagation.

The *35S::CrWRKY57* overexpression construct and EV were introduced into *N. tabacum* to generate transgenic tobacco using the leaf disc method [68]. Drought tolerance assays were conducted using T2 generation plants. *Actin* and *ubiquitin* were used as internal reference genes for *Citrus* and tobacco, respectively, in regular PCR and RT-qPCR confirmation of transgenic plants.

### Physiological measurements

RWL was calculated as the ratio of the lost weight after dehydration to the initial weight. EL was measured according to Peng *et al.* [69]. MDA and H_2_O_2_ contents, as well as anti-O_2_^·–^ capacity, were measured using corresponding assay kits (Nanjing Jiancheng Bioengineering Institute, Nanjing, China).

### Histochemical staining of ROS in leaves and dead cells in roots

*In situ* accumulation of hydrogen peroxide (H_2_O_2_) and superoxide (O_2_^·–^) in leaves was histochemically stained by 1 mg/mL DAB and NBT for 12 hours, respectively, followed by decolorization in 80% ethanol [70, 71]. PI staining of tobacco root tip was performed according to Bureau *et al.* [72] with minor modifications. Briefly, 3 days after germination on an MS medium, tobacco seedlings were moved to the MS medium supplemented with 0.4 M mannitol to mimic dehydration for another 3 days. Subsequently, roots of 6-day-old tobacco seedlings continuously growing on either the MS medium (as control) or MS + 0.4 M mannitol were stained with 10 mg/L PI for 8 minutes and then washed with 0.01 M PBS buffer (pH = 7.4). Longitudinal sections of the PI-stained root tips were imaged using a Leica TCS SP8 confocal microscope system with an excitation wavelength of 535 nm and emission fluorescence collected at 615 nm for PI.

### Generation of VIGS plants and root scanning

To generate VIGS plants, fragments of *CrWRKY57*, *CrABF3* or *CrCYCD6;1* were inserted between BamHI and SmaI sites of the tobacco rattle virus (TRV)-based vector 2 (pTRV2). The recombinant vectors and the pTRV1 + pTRV2 (control) were separately transformed into GV3101. Preparation of the bacterial infection suspensions was performed as described by Dai *et al.* [16]. After agroinfiltration, Sanhu seeds were sown in soil pots and placed in a growth chamber (28°C; 16-hour light/8-hour dark). After 45–60 days, control, TRV-*CrWRKY57*, TRV-*CrABF3*, and TRV-*CrCYCD6;1* plants were screened by PCR and RT-qPCR. Roots were scanned with the WinRHIZO™ LA2400 Scanner (Regent Instruments, Canada). Primary root length and lateral root number were measured using ImageJ.

### Measurement of ABA

Tender leaves (50 mg) from 1-year-old *CrWRKY57*-OE lemon and *CrWRKY57*-RNAi Sanhu as well as their corresponding controls were collected. Then the fresh tissue was homogenized in liquid nitrogen and extracted with methanol/water/formic acid (15:4:1, v/v/v) containing 10-μL d6-ABA internal standard (100 ng/mL), followed by vortexing (10 minutes) and centrifugation (12 000 rpm, 4°C, 5 minutes). The supernatant was analyzed by LC–MS/MS System (Sciex QTRAP^®^ 6500+) using a C18 column (Waters ACQUITY UPLC HSS T3) and 0.04% acetic acid/acetonitrile gradient (Nanjing Convinced-test Technology Co., Ltd, Nanjing, China).

### BiFC assay

The constructs of pSPYNE-35S-*CrWRKY57* and pSPYCE-35S-*CrABF3* were separately transformed into GV3101. Following transformation, the two strains were co-infiltrated into leaves of 50-day-old tobacco plants (*N. benthamiana*) as described by Liu *et al.* [73]. The agroinfiltrated tobacco plants were then allowed to grow for 48 hours before the yellow fluorescence was examined using the Leica DM6 B microscope system.

### Co-IP assay

The coding sequences of *CrABF3* and *CrWRKY57* were inserted between KpnI and XbaI sites of pCAMBIA1300-GFP and pCAMBIA1300-35S-3*FLAG, respectively. The two constructs were injected into the abaxial side of 50-day-old tobacco leaves (*N. benthamiana*). After 48 hours of injection, leaves excluding the main vein were ground in liquid nitrogen, and total protein was extracted in Co-IP buffer. Subsequently, the proteins were purified with Anti-FLAG M2 Affinity Gel and used for western blot using ANTI-FLAG and ANTI-GFP antibodies (Sigma-Aldrich, Shanghai, China).

### Yeast two-hybrid

The Y2H assay was performed using a Matchmaker™ Gold Yeast Two-Hybrid System (Clontech, USA). The pGBKT7-*CrWRKY57* and pGADT7-*CrABF* plasmids were co-transformed into the Y2HGold strain cells and grown on SD/−Leu/−Trp (DDO), SD/−Leu/−Trp/-His/−Ade (QDO) and QDO + X-α-gal (QDO/X) agar media plates at 28°C for 3 days.

### Yeast one-hybrid

Fragments of the *CrCYCD6;1* promoter containing W-box or ABRE element were fused to the pAbAi vector as bait pW1 (−423 to −304 bp), pW2 (−303 to −184 bp), pA1 (−664 to −544 bp), and pA2 (−543 to −424 bp), respectively. Fragments of *CrABF1* and *CrABF3* containing W-box were also inserted into the pAbAi vector. The full-length ORF of *CrWRKY57* or *CrABF3* without the stop codon was fused to pGADT7 to generate prey vectors. The baits and prey constructs were co-transformed into yeast strain Y1HGold following the instructions provided by Yeastmaker™ Yeast Transformation System 2 (Clontech). The yeast strains harboring pAbAi-p53 and pGADT7-p53 served as positive controls, whereas those harboring baits and empty pGADT7 served as negative controls. The yeast cells were cultured on an SD/−Leu/-Ura medium supplemented with 0, 100, or 200 ng/mL Aureobasidin A (AbA).

### Electrophoretic mobility shift assay

*CrWRKY57* and *CrABF3* CDSs were separately cloned into the pDEST-HisMBP vector with a His tag. The two fusion proteins were induced at 37°C for 6–8 hours using 0.5 mM β-d-1-thiogalactopyranoside (IPTG) and purified using His-Tagged Protein Purification Kit (CWBIO, China). Probes containing a W-box (TTGACC) element and a mutant W-box (TTTTCC) element from the *CrCYCD6;1* promoter were synthesized and labeled with biotin by Tsingke Biological Technology (Beijing, China). Probes containing an ABRE (ACGTG) element and a mutated ABRE element (TTGTG) were also synthesized and labeled. The unlabeled probe was used as the competitor. Gel-shift assays were performed using the LightShift Chemiluminescent EMSA Kit in accordance with the manufacturer’s protocol (Thermo Fisher Scientific).

### Histochemical GUS assay

The 1036-bp *CrCYCD6;1* promoter was inserted into the DX2181 vector containing a *GUS* reporter gene, which was used as the reporter. The pCAMBIA1300-eGFP EV and vectors ligated with the ORFs of *CrWRKY57* or *CrABF3* were used as the effectors. *Agrobacterium* injection solution was prepared and injected into *N. benthamiana* leaves, which were cultured in the dark for 3 days, and then incubated in the X-Gluc solution at 37°C for 12 hours. Chlorophyll was removed using 75% (v/v) ethanol, and then the leaves were photographed. The blue area indicating GUS activity was quantified using ImageJ.

### Dual luciferase assay

The full-length ORFs of *CrWRKY57* and *CrABF3* were separately cloned into pGreen II 62-SK to serve as effectors. The *CrCYCD6;1* promoter was inserted into pGreen II 0800-LUC as the reporter. The effector and reporter constructs were co-transformed into *N. benthamiana* leaves. The combination of empty pGreen II 62-SK and reporter was used as the control. After a 48-hour postinfiltration, firefly luciferase (LUC) and *Renilla* luciferase (REN) activities were measured using the Dual-Luciferase^®^ Reporter Assay System (Promega, USA) on a Spark 10M microplate reader (Tecan, Switzerland). Images were captured using the NightSHADE LB 985 imaging system (Berthold, Germany).

### Statistical analysis

Statistical analyses were performed using GraphPad Prism 9.0. All data are shown as means ± standard error (SE). Statistical significance was determined using one-way analysis of variance (ANOVA, *P* < 0.05) or Student’s *t*-test (^*^P < 0.05, ^**^P < 0.01, ^***^P < 0.001).

## Supplementary Material

Web_Material_uhaf158

## Data Availability

All relevant data in this study can be found in the article and its supporting files.
