# Supplementary material for: CrWRKY57 and CrABF3 cooperatively activate CrCYCD6;1 to modulate drought tolerance and root development
Source: Hortic Res. 2025 Jun 20;12(9):uhaf158. doi: 10.1093/hr/uhaf158 (PMC12373975; doi:10.1093/hr/uhaf158)
Supplement: Web_Material_uhaf158 [file web_material_uhaf158.docx]

**Supplemental Figure S1.**

**
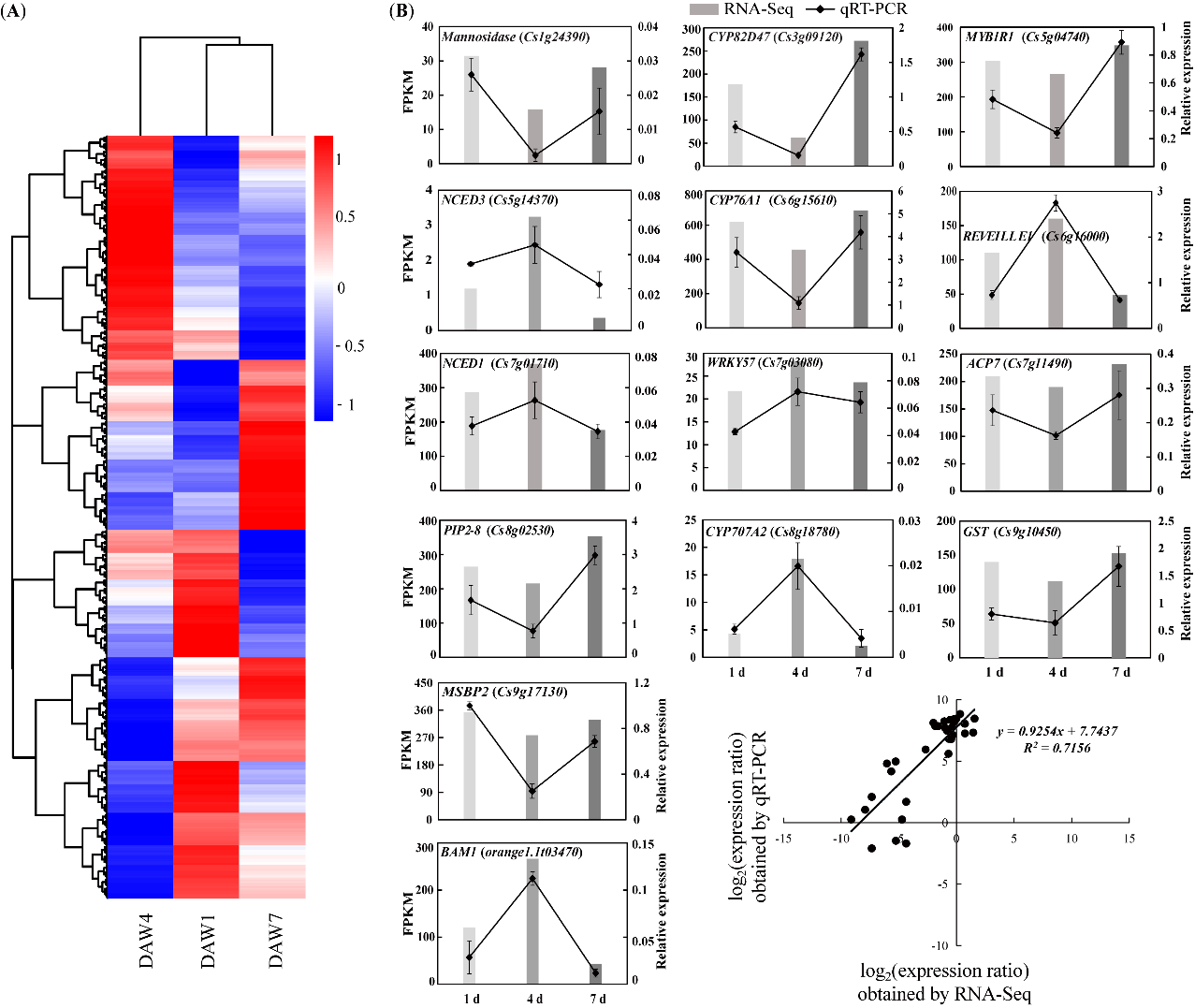
**

**Supplemental Figure S1.** Transcriptome profiling and RT-qPCR verification of drought-stressed Sanhu. (**A**) Heat map of differentially expressed genes (DEGs) in Sanhu leaves after water withholding for 1, 4 and 7 days (DAW1, 4 and 7). (**B**) RT-qPCR validation of the relative expression levels of 14 DEGs. The strong correlation between RT-qPCR and RNA-seq results confirms the reliability of the RNA-seq data.

**
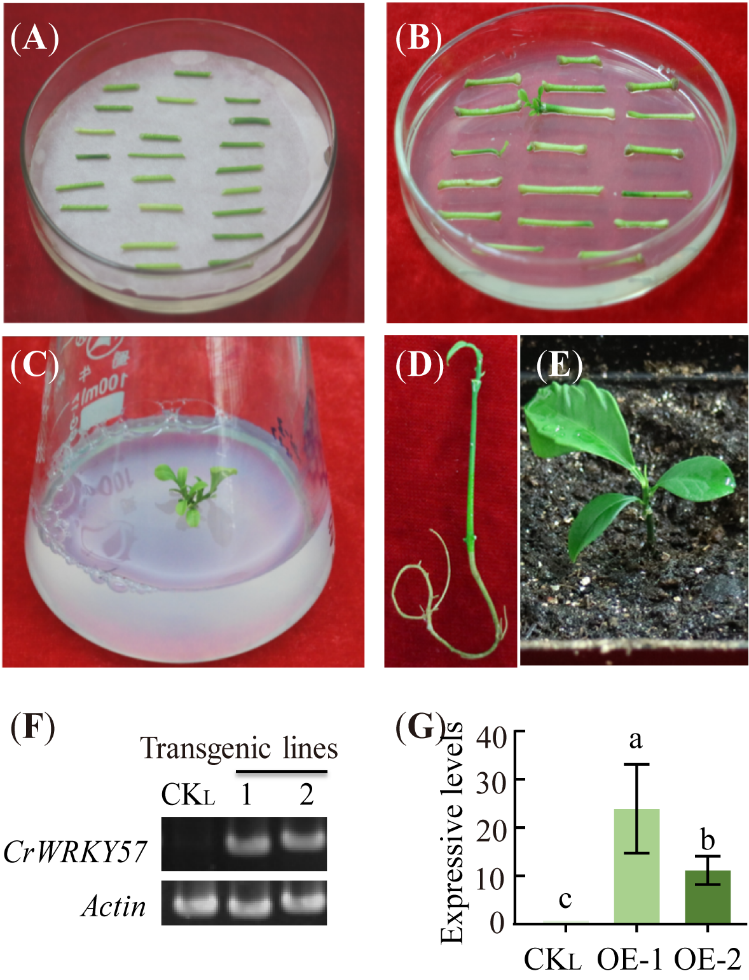
**

**Supplemental Figure S2.** *Agrobacterium tumefaciens*-mediated generation and micrografting of transgenic lemon lines overexpressing *CrWRKY57*. *Poncirus trifoliata* was used as rootstock.

**
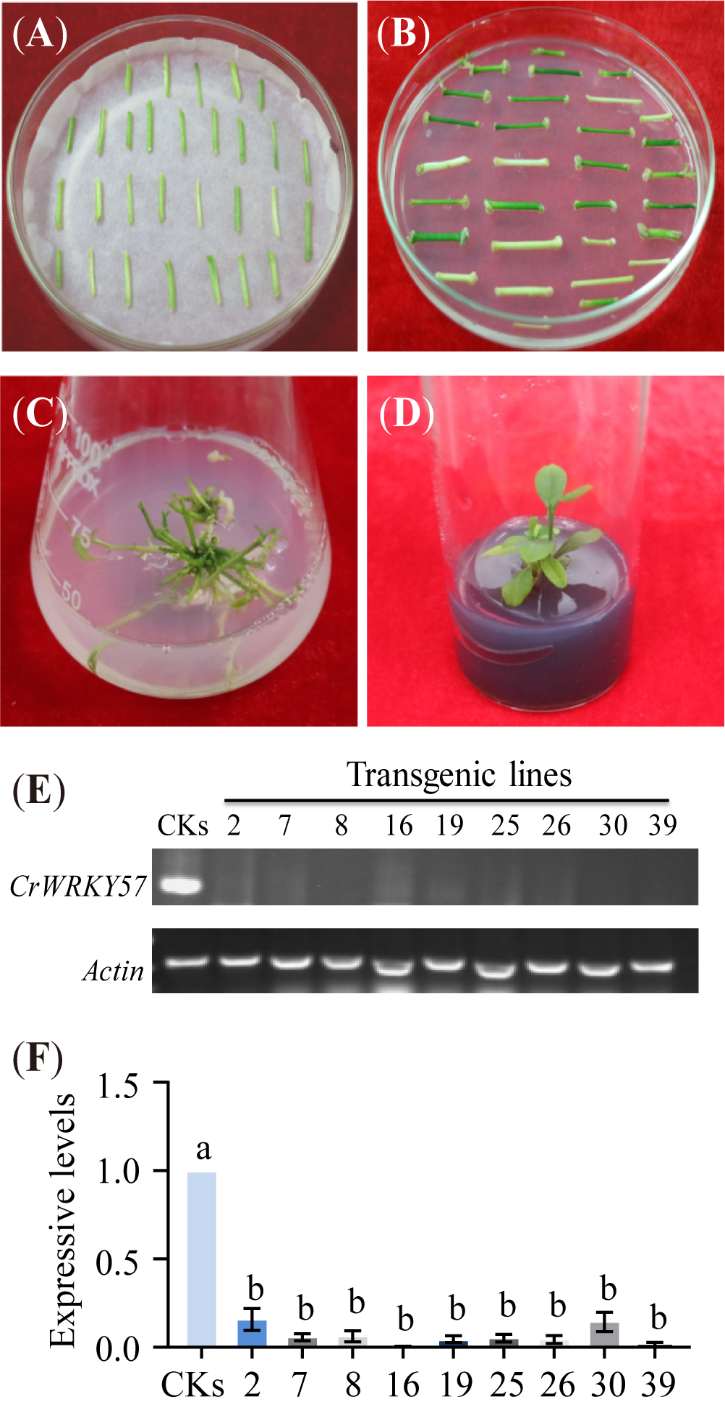
**

**Supplemental Figure S3.** *Agrobacterium tumefaciens*-mediated generation and identification of *CrWRKY57*-RNA interference Shanhu lines.

**
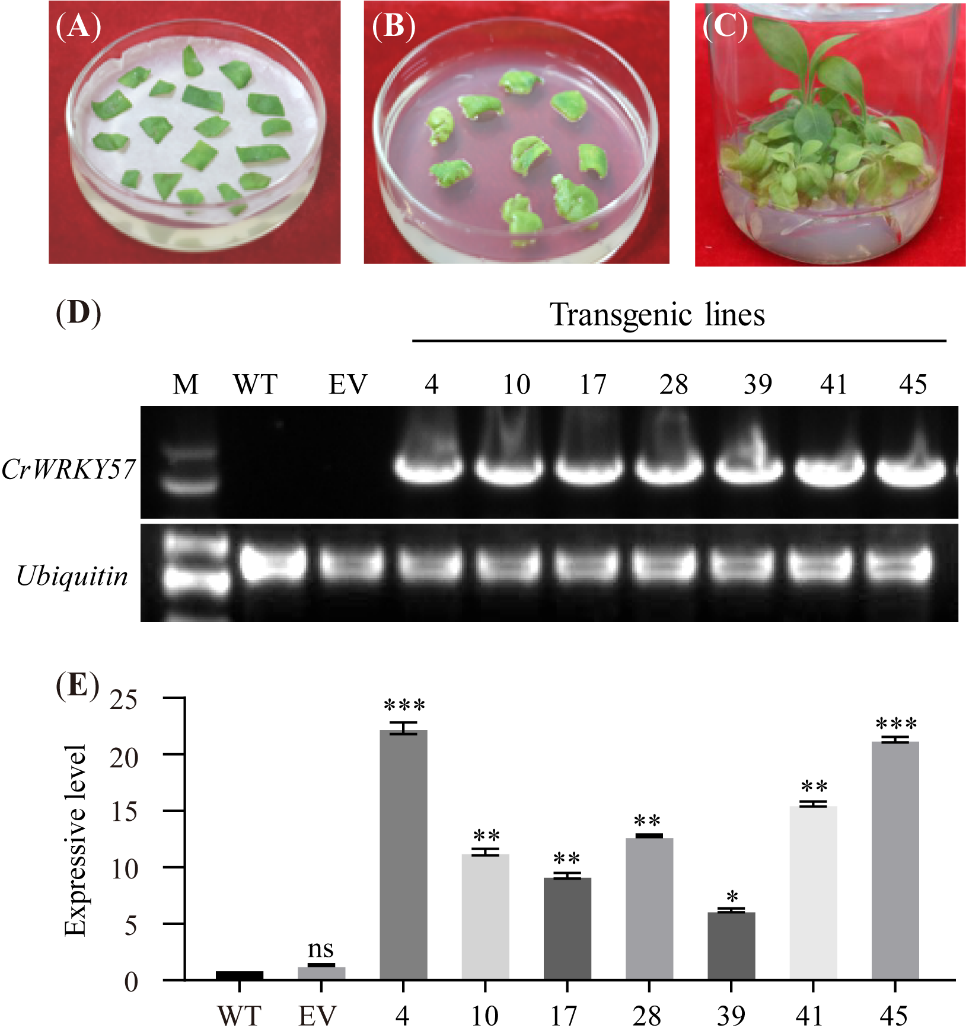
**

**Supplemental Figure S4.** *Agrobacterium tumefaciens*-mediated transformation, plant regeneration and identification of *CrWRKY57*-overexpression tobacco (*Nicotiana tabacum*).

**
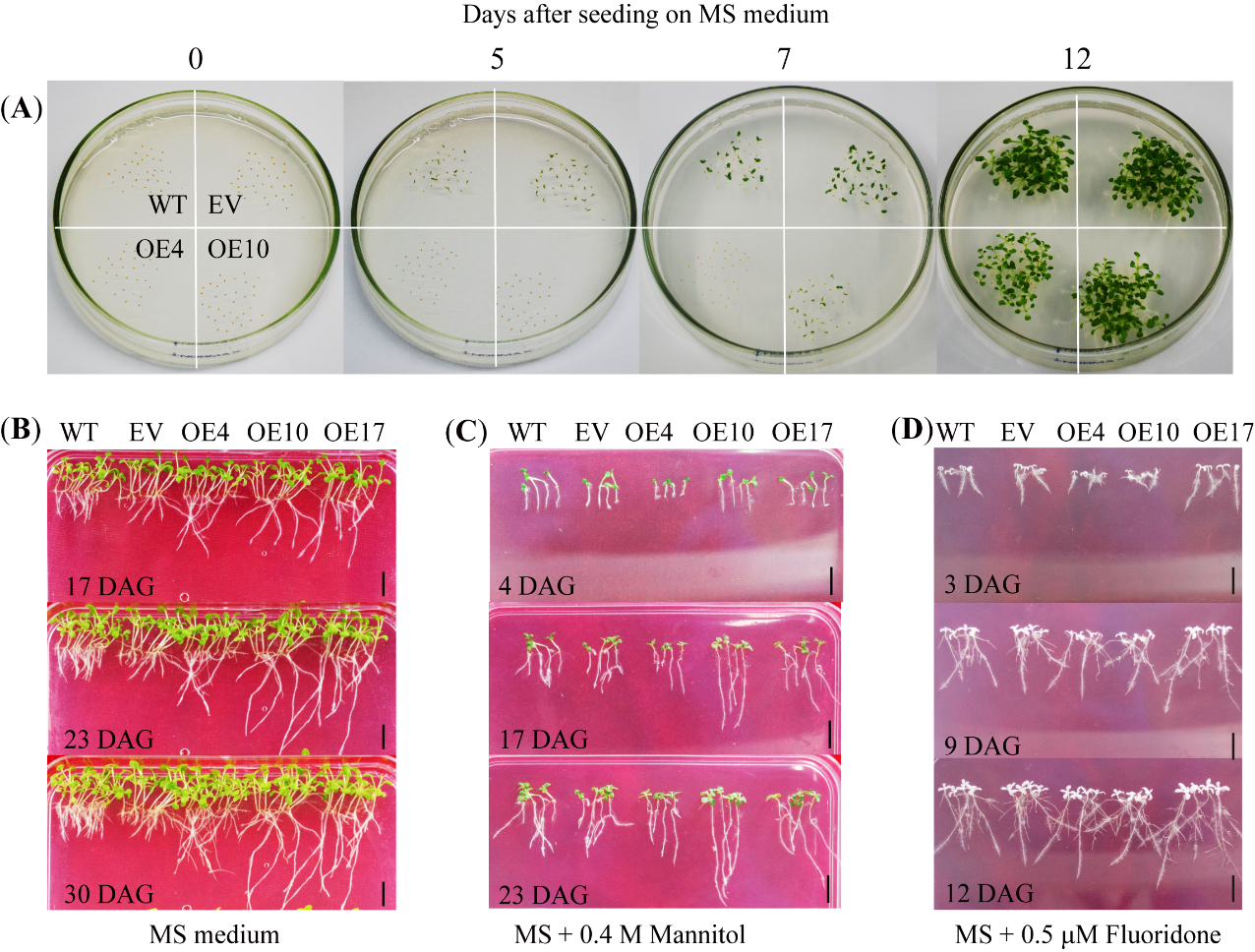
**

**Supplemental Figure S5.**  Overexpressing *CrWRKY57* in tobacco (*N. tabacum*) alters root development. (**A**) Germination of wild type (WT), empty vector line (EV), and *CrWRKY57*-overexpression tobacco lines (OE4, OE10) on MS media. OE17 also germinated later than the controls (not shown due to space limitations). (**B**-**D**) Images of WT, EV, OE4, OE10, and OE17 on MS media (**B**), MS + 0.4 M mannitol media (**C**), MS + 0.5 μM fluoridone media (**D**) after germination. DAG, days after germination. Scale bars, 1 cm.

**
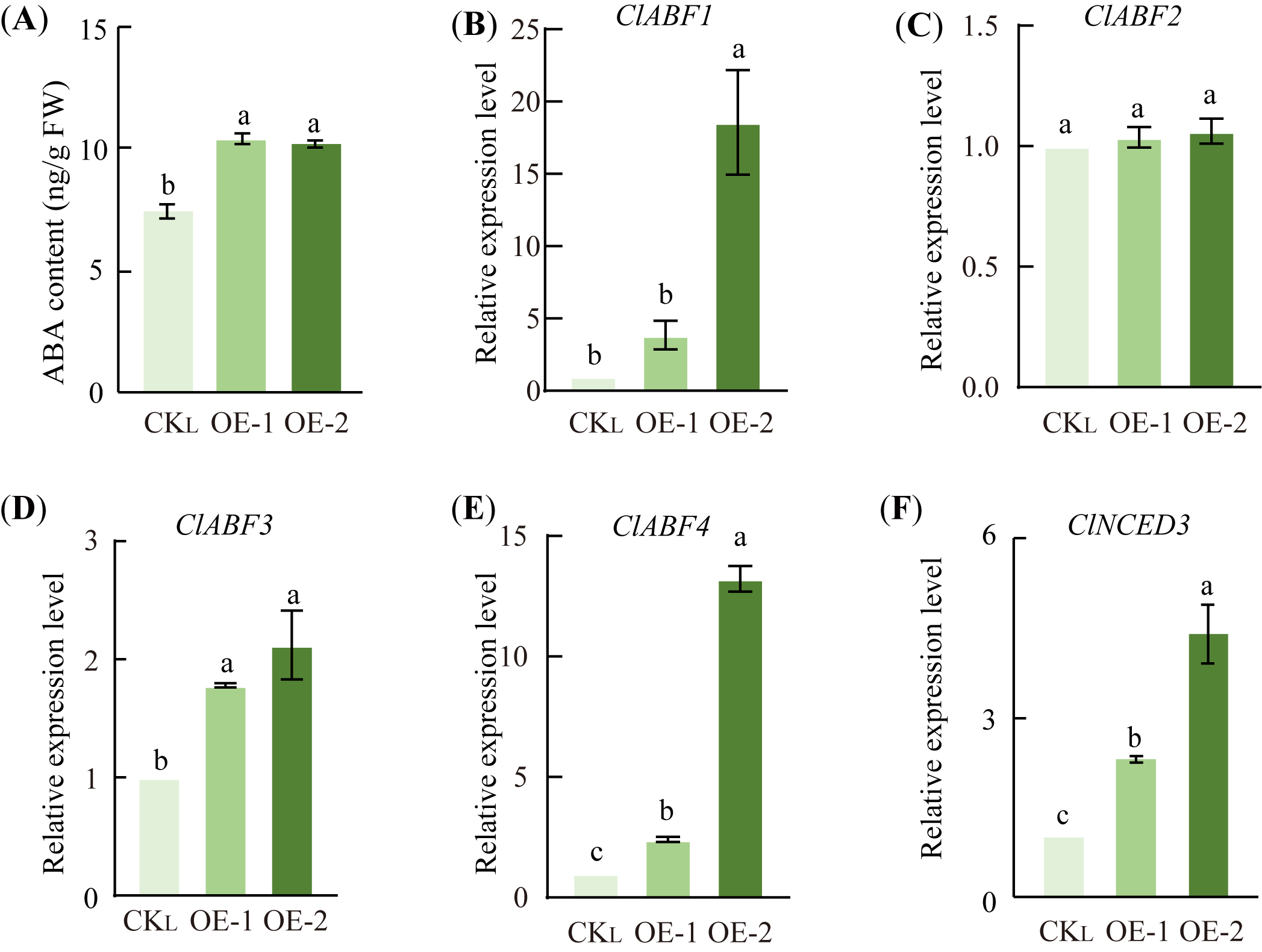
**

**Supplemental Figure S6.** ABA content and RT-qPCR analysis of ABA-related genes in *CrWRKY57*-overexpression lemon lines. ABA content (**A**) and RT-qPCR analysis of *ABF1* (**B**), *ABF2* (**C**), *ABF3* (**D**), *ABF4* (**E**), *NCED3* (**F**) in *CrWRKY57*-overexpression lemon lines (OE-1 and OE-2) and its control (CK_L_). Different letters above columns indicated significant differences (*P* < 0.05).

**
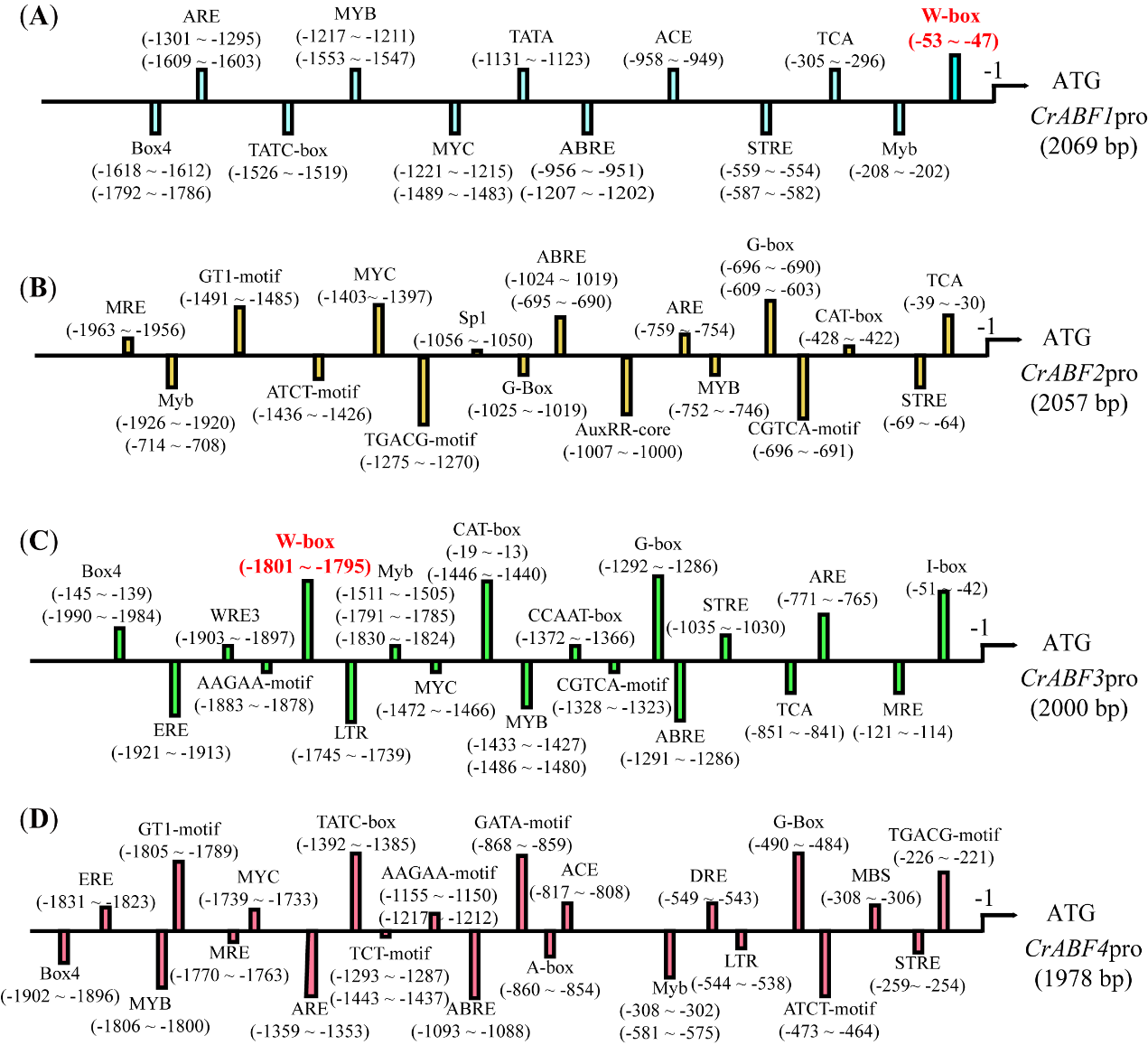
**

**Supplemental Figure S7.** Schematic diagram of promoter regions of *CrABF1*-*4*. W-box *cis*-element was marked in red.

**Supplemental Figure S8.**

**
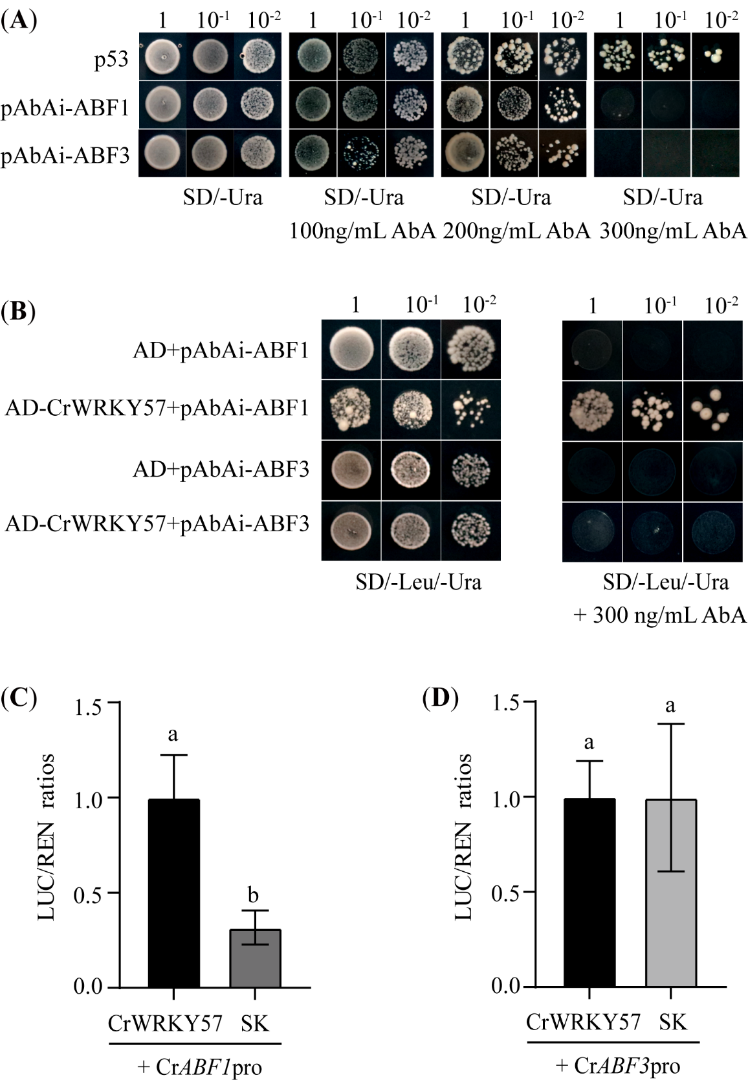
**

**Supplemental Figure S8.** CrWRKY57 directly binds to the *CrABF1* promoter and inhibits its transcription, not *CrABF3*. (**A**) Yeast one-hybrid (Y1H) aureobasidin A (AbA) concentration screening. (**B**) Y1H assay. Growth of yeast cells co-transformed with prey (pGADT7-CrWRKY57) and baits (pAbAi-*ABF1*pro and pAbAi-*ABF3*pro) on SD/-Ura/-Leu medium added with 300 ng/mL AbA. Positive control, pAbAi-p53 and pGADT7-p53; Negative control, bait + pGADT7. (**C**, **D**) Dual luciferase assay. Co-transformation of effectors (SK, empty pGreen II 62-SK; CrWRKY57, pGreen II 62-SK ligated with CrWRKY57) and reporter (pGreen II 0800-LUC ligated with *ABF1*pro or *ABF3*pro) into *N. benthamiana* leaves. The LUC/REN ratios of SK and reporter was set as 1 for normalization.

**
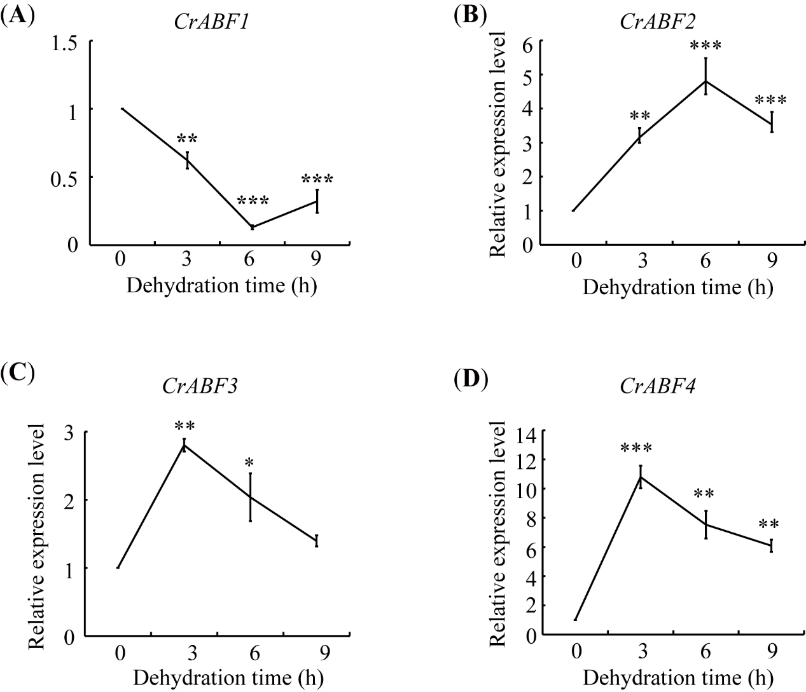
**

**Supplemental Figure S9.** RT-qPCR analysis of *CrABF1*-*4* expression level under dehydration. *, *P* < 0.05; **, *P* < 0.01; ***, *P* < 0.001.

**
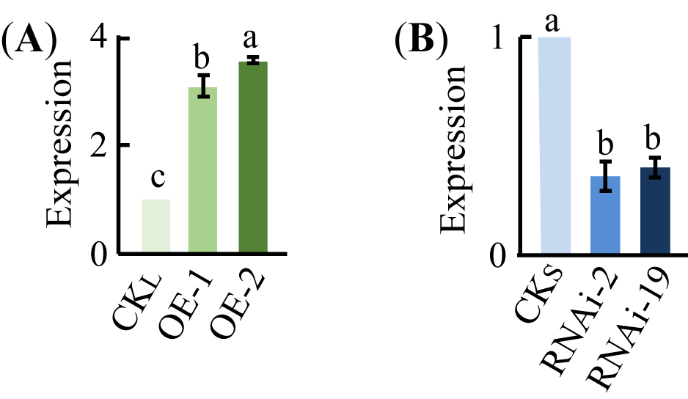
**

**Supplemental Figure S10.** RT-qPCR analysis of *CrCYCD6;1* expression level in *CrWRKY57*-overexpression lemon lines and *CrWRKY57*-RNAi Sanhu lines compared to their respective controls. Different letters above bars indicate significant differences (*P* < 0.05).
